# Recurrent Bilateral Strokes in a Patient Treated With Sipuleucel-T for Prostate Cancer

**DOI:** 10.7759/cureus.14596

**Published:** 2021-04-20

**Authors:** Jashan Gill, Hafiz Muhammad Jeelani, Sonika Prasad, Nayha Tahir

**Affiliations:** 1 Internal Medicine, Northwestern Medicine McHenry Hospital, Rosalind Franklin University of Medicine and Science, McHenry, USA; 2 Internal Medicine, Chicago Medical School, Rosalind Franklin University of Medicine and Science, McHenry, USA; 3 Internal Medicine, Northwestern Medicine McHenry Hospital, Rosalind Franklin University of Medicine and Science, Mchenry, USA

**Keywords:** sipuleucel-t, castration resistant prostate cancer, embolic stroke of undetermined source

## Abstract

Sipuleucel-T is approved by the US Food and Drug Administration (FDA) for the treatment of castration-resistant prostate cancer (CRPC). Herein, we present a patient with recurrent bilateral embolic stroke who was on sipuleucel-T therapy for CRPC. Laboratory and imaging data didn’t reveal any source of embolic stroke. A focused history disclosed that the patient received two doses of sipuleucel-T before the first stroke and was advised not to receive his third dose. He reported no other episode of stroke at the six-month follow-up. This case highlights the importance of identifying sipuleucel-T as a potential cause of embolic stroke if the source is not detectable, as discontinuing the therapy can be beneficial. Physicians should evaluate patients for risk of stroke before starting the therapy to prevent future strokes.

## Introduction

The treatment of prostate cancer has been revolutionized by sipuleucel-T, which has been shown to reduce the risk of death by 22% [1]. It is a personalized cellular immunotherapy synthesized in-vitro by harboring a patient’s dendritic cells and priming them against tumor antigens specific to prostate carcinoma. In clinical trials, adverse events most commonly occurred after the second dose of infusion. Cerebrovascular events were reported in preclinical and post-marketing trials, leaving the US Food and Drug Administration (FDA) to continue monitoring for further events despite statistically non-significant results. To our knowledge, there is no case of embolic stroke associated with sipuleucel-T therapy reported in the post-marketing period. We report this case in follow-up to the FDA’s monitoring for sipuleucel-T-associated adverse events, as our patient presented with recurrent bilateral embolic stroke after the second dose in the absence of any other identifiable source of embolism. 

## Case presentation

**Figure 1:**
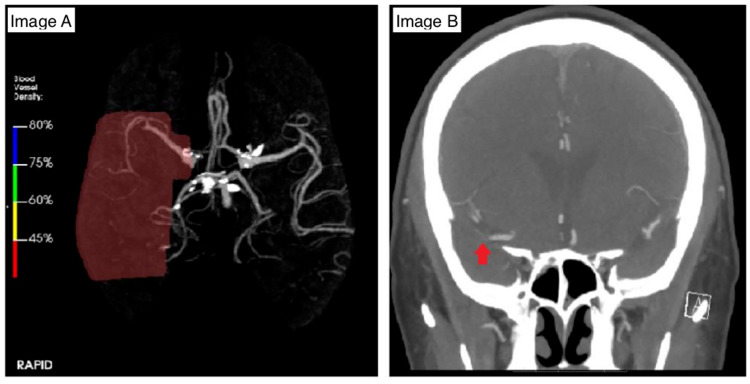
Computed tomography angiography of the patient's brain. Image A: diminished blood vessel density in the right middle cerebral artery (MCA) distribution (highlighted red area) with low blood vessel density due to occlusion of the major M2 and M3 branches; Image B: right-sided M2 branch occlusion (red arrow).

A 77-year-old Caucasian male with a past medical history of stage IV prostate cancer on sipuleucel-T therapy presented with left-sided weakness, which was present when he awoke from sleep. On presentation, vital signs were stable. The physical examination was significant for left-sided hemiparesis, homonymous hemianopia, and hemineglect. The rest of the physical examination was unremarkable. Initial laboratory workup, including complete blood count and comprehensive metabolic profile, was within normal limits. Computed tomography angiography (CTA) of the brain revealed ischemic stroke involving the right middle cerebral artery with occlusion of M2 and M3 branches (Figure 1). 

The patient was not a candidate for tissue plasminogen activator (tPA) as the onset of symptoms was unknown; therefore, he underwent emergent cerebral angiography with mechanical thrombectomy. On day one post-thrombectomy, magnetic resonance imaging (MRI) of the brain showed scattered foci of acute ischemia throughout the bilateral posterior circulation and left middle cerebral artery distribution, suggestive of embolic stroke. Transthoracic echocardiogram was negative for thrombus, patent foramen ovale, or any valvular abnormalities. The patient's neurological symptoms significantly improved over the hospital course. He was discharged to a rehabilitation facility on aspirin 81 mg, atorvastatin 40 mg daily, and a cardiac event recorder. A review of data from the recorder showed normal sinus rhythm throughout the 30 days of records.

**Figure 2:**
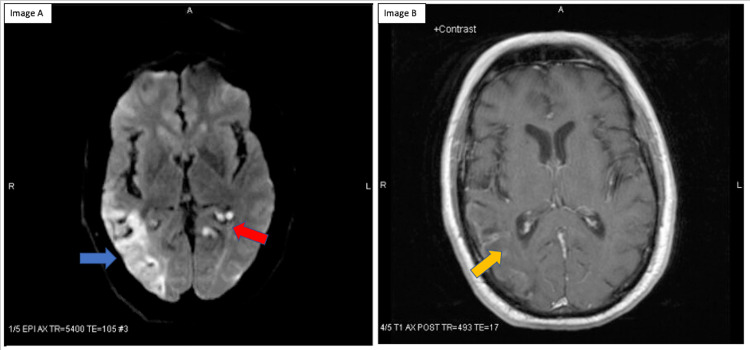
MRI brain of the patient. Image A: Diffusion-weighted imaging demonstrating multiple strokes in the left medial thalamus (red arrow), subacute right temporal-occipital infarct (blue arrow); Image B: T1 post TR displaying luxury perfusion of the subacute infarct.

Two weeks later, the patient returned to the hospital with bilateral leg weakness for 2 hours. MRI brain showed multiple new small acute infarcts throughout the bifrontal white matter, left medial thalamus, and left occipital lobe (Figure 2). There was also a subacute right temporal, occipital infarct consistent with the prior stroke displaying luxury perfusion.

Further workup involving lower extremity doppler and a transesophageal echocardiogram was unrevealing for a cardioembolic source of stroke. However, it was discovered that the patient received two doses of sipuleucel-T; the second dose was given one week before his initial stroke episode. His symptoms improved during the hospital course. Given the negative ischemic stroke workup, sipuleucel-T was considered as the potential etiology. Therefore, the patient was recommended not to receive further infusion and was discharged to an acute rehab facility.

The patient reported no subsequent episodes of ischemic stroke at the six-month follow-up. At this time, a workup for a hypercoagulability disorder was pursued. Blood tests were ordered for beta-2 glycoprotein I antibodies (IgG, IgA, IgM), cardiolipin antibodies (IgG, IgM, IgA), protein c activity, protein s activity, prothrombin (factor II) mutation, and factor V Leiden, all of these returned negative.

## Discussion

Sipuleucel-T is approved by the FDA as the first cancer vaccine to treat asymptomatic or minimally symptomatic castration-resistant prostate cancer [2]. It is an autologous cellular immunological agent. Although not well defined, the generation of a strong, persistent antigen-specific humoral and T-cell immune response by activated antigen-presenting cells is the proposed mechanism of action, which targets prostatic acid phosphatase, a highly expressed antigen in most prostate cancer cells [3,4].

The most common adverse events (AEs) are infusion reactions such as chills, fatigue, fever, respiratory events (hypoxia, bradycardia, and bronchospasm), and headaches, particularly after the second infusion dose, as in this case. The drug is administered for three doses at two weeks intervals [3,5]. The cerebrovascular events (CVEs) were observed in 3.5% of the sipuleucel-T group compared to 2.6% based on the pooled data from four randomized placebo control trials (Table 1), which led the FDA to recommend a post-marketing study; however, the results were not statistically significant [1,5-7].

**Table 1:** Incidence of cerebrovascular events in clinical trials of sipuleucel-T.

Study	Year published	Participants (n)	The reported incidence of cerebrovascular events
Higano CS et al [6]	2009	n=147 (sipuleucel-T), n=78 (control)	11 of 147 (7.5%) participants in the sipuleucel-T arm vs 2 of 76 (2.6%) in the control arm
Kantoff PW et al [1]	2010	n=147 (sipuleucel-T), n=78 (control)	8 of 338 (2.4%) participants in the sipuleucel-T arm vs 3 of 168 (1.8%) in the control arm
Higano CS et al. (PROCEED) [8]	2019	n=1976 (sipuleucel-T)	54 of 1976 (2.8%) patients treated with sipuleucel-T

The Provenge® [Dendreon Pharmaceuticals, LLC, Seal Beach, USA] Registry for Observation, Collection, and Evaluation of Experience Data (PROCEED) trial was conducted in the follow-up. They concluded that the causal relationship between CVEs and sipuleucel-T was unclear, but subgroup analyses determined that older patients with CVE risk factors had higher rates of CVEs [8]. A post-marketing safety assay conducted by Dores et al. revealed 17 cases of stroke (six embolic, seven hemorrhagic, and six ischemic), and four patients reported strokes occurring after the second infusion [9]. The results were reassuring, yet US FDA continues to monitor reports of AEs, including CVEs from sipuleucel-T infusion. Our patients experienced two embolic strokes after the second infusion with the maximal neurologic deficit at onset followed by improvement in symptoms, a typical presentation for an embolic phenomenon [10,11].

Although the diagnosis can be confirmed by MRI of the brain, CTA or magnetic resonance angiography of head and neck, transesophageal echocardiogram, event monitor for arrhythmias, and hypercoagulability workup are essential to rule out other etiologies of embolic strokes, particularly cardiovascular causes, to correlate stroke with sipuleucel-T. All of these diagnostic studies were pursued in our patient; however, a source was not determined. This led us to postulate that sipuleucel-T played a role in the pathogenesis because of the reports from the initial clinical trials. Treatment is the same as in other cases of embolic stroke, along with discontinuations of the offending agent. Despite our investigation, it is important to consider that embolic strokes remain undetermined in 33% of cases [12]. Hypercoagulability in the setting of malignancy is a possibility in our case; however, the outpatient workup for a coagulation disorder was negative.

## Conclusions

Cumulative data from randomized trials have suggested that men with prostate cancer treated with sipuleucel-T have a higher frequency of strokes compared to those receiving a placebo. Although this finding was not statistically significant, these trials were not designed to detect differences in stroke incidence related to sipuleucel-T treatment. Furthermore, it is prudent to rule out all other possible etiologies of stroke before considering sipuleucel-T as the cause. Evaluation of patients for stroke risk factors before initiation of therapy may help prevent stroke, and physicians should consider this in their differential diagnosis, especially in patients presenting with stroke symptoms and are on sipuleucel-T therapy.
